# Deciphering the complex leaf transcriptome of the allotetraploid species *Nicotiana tabacum*: a phylogenomic perspective

**DOI:** 10.1186/1471-2164-13-406

**Published:** 2012-08-17

**Authors:** Aureliano Bombarely, Kieron D Edwards, Juan Sanchez-Tamburrino, Lukas A Mueller

**Affiliations:** 1 Boyce Thompson Institute for Plant Research, Tower Road, Ithaca, NY, 14853-1801, USA; 2 Advanced Technologies (Cambridge Ltd), 210 Cambridge Science Park, Milton Road, Cambridge, CB4 0WA, UK

**Keywords:** *Nicotiana tabacum*, Phylogenomic, Polyploid, Sequence assembly, Homeolog identification, Tree topology, Transcriptome, Tobacco, Next generation sequencing, 454

## Abstract

**Background:**

Polyploidization is an important mechanism in plant evolution. By analyzing the leaf transcriptomes taken from the allotetraploid *Nicotiana tabacum* (tobacco) and parental genome donors, *N. sylvesteris* (S-Genome) and *N. tomentosiformis* (T-Genome), a phylogenomic approach was taken to map the fate of homeologous gene pairs in this plant.

**Results:**

A comparison between the genes present in the leaf transcriptomes of *N. tabacum* and modern day representatives of its progenitor species demonstrated that only 33% of assembled transcripts could be distinguished based on their sequences. A large majority of the genes (83.6% of the non parent distinguishable and 87.2% of the phylogenetic topology analyzed clusters) expressed above background level (more than 5 reads) showed similar overall expression levels. Homeologous sequences could be identified for 968 gene clusters, and 90% (6% of all genes) of the set maintained expression of only one of the tobacco homeologs. When both homeologs were expressed, only 15% (0.5% of the total) showed evidence of differential expression, providing limited evidence of subfunctionalization. Comparing the rate of synonymous nucleotide substitution (Ks) and non-synonymous nucleotide substitution (Kn) provided limited evidence for positive selection during the evolution of tobacco since the polyploidization event took place.

**Conclusions:**

Polyploidization is a powerful mechanism for plant speciation that can occur during one generation; however millions of generations may be necessary for duplicate genes to acquire a new function. Analysis of the tobacco leaf transcriptome reveals that polyploidization, even in a young tetraploid such as tobacco, can lead to complex changes in gene expression. Gene loss and gene silencing, or subfunctionalization may explain why both homeologs are not expressed by the associated genes. With Whole Genome Duplication (WGD) events, polyploid genomes usually maintain a high percentage of gene duplicates. The data provided little evidence of preferential maintenance of gene expression from either the T- or S-genome. Additionally there was little evidence of neofunctionalization in *Nicotiana tabacum* suggesting it occurs at a low frequency in young polyploidy.

## Background

Polyploidy occurs either through the combination of two or more genomes from different parents (alloploidization), or the multiplication of an endogenous genome (autoploidization). The majority of flowering plants have undergone polyploidization (whole genome duplication events [WGD]) during their evolutionary history, suggesting that it provides a mechanism that can increase the fitness of an organism [1], possibly through heterosis [2]. Two WGD events are dated to have occurred before the diversification of extant seed plants and extant angiosperms [3]. Analysis of the *Arabidopsis thaliana* genome supports three more recent WGD events (named γ, β and α). Evidence from investigations on the genome sequences of *Vitis vinifera* and *Medicago truncatula*[4-6], suggests that the first, or γ event, extends to all the core-eudicots and many other plant species. Polyploidization is relatively common in agricultural and commercial species, such as wheat (*Triticum aestivum*), potato *(Solanum tuberosum),* coffee (*Coffea arabica*) and cotton (*Gossypium hirsutum*), indicating that this evolutionary mechanism may be important in plant domestication.

Polyploidization involves complex genetic and epigenetic process and genome duplication is often followed by changes in gene expression and gene loss [7-14]. Complementary hypotheses that explain this phenomena suggest that either selection is based on absolute gene dosage, or relative gene dosage (dosage balance) [15]. The absolute gene dosage hypothesis states that gene networks have balanced states of interaction that are critical for proper function and any disturbances on the network’s stoichiometry of interaction are not optimal for plant survival. The relative dosage hypothesis argues that a gene product can have multiple interactions that may assist in the survival of the plant, upon which selection is based.

Duplicated genes generated by polyploidization events are referred to as homeologs. The fate of homeologous genes can be divided in four general categories; conservation or redundancy, nonfunctionalization (gene function loss for one copy), subfunctionalization (partitioning ancestral functions/expression patterns between duplicated genes) and neofunctionalization (evolution of a gene copy to a new function) [16,17]. The relative ratio of these gene fates may differ between species.

*Nicotiana* species are excellent models for investigating plant polyploidization. Approximately 40% of *Nicotiana* genera are allotetraploids [18,19]. With an estimated age of 0.2 Myr [20], *Nicotiana tabacum* is a relatively young allotetraploid originating through the hybridization of *Nicotiana sylvesteris* (maternal, S-genome donor) [21,22] and *Nicotiana tomentosiformis* (paternal, T-genome donor) [21,23]. Extensive studies within the *Nicotiana* genus, and specifically within *N. tabacum* including the first generation of a synthetic *N. tabacum*, have revealed a complex landscape for polyploid genome evolution [24]. Many evolutionary changes in the tobacco genome have been elucidated. They include evidence for an early genomic shock [25], a great increase in the frequency of heterozygosity and T-genome repeat losses leading to genome size reduction [26,27]. Other evolutionary events, such as intergenomic translocations [24,28,29] and epigenetic patterns of 45S rDNA expression have been characterized as well [30]. In addition, gene expression studies of *N. tabacum* have been performed using microarrays [31], although the technology may have limited ability to distinguish between homeologs.

This study presents a characterization of the *N. tabacum* transcriptome constructed from an evolutionary perspective by combining a Next Generation Sequencing (NGS) and expression analysis with a phylogenetic approach applied on a genomic scale.

## Results

### Transcriptome assembly and annotation for a polyploid species

A set of expressed sequence tags (ESTs) was generated from leaves of *N. tabacum* and modern day representatives of its progenitor species *N. sylvesteris* and *N. tomentosiformis*. Test assemblies of the *N. sylvesteris* ESTs were generated using several programs (see Materials and Methods). GsAssembler produced longer contigs and was significantly faster than the other assembly programs (data not shown) so it was selected for further optimization.

**Figure 1:**
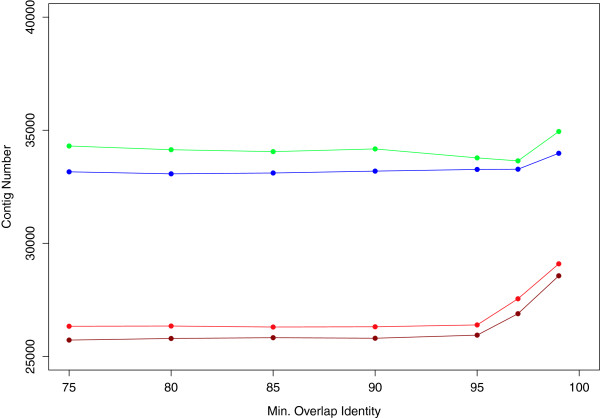
***Nicotiana *EST assemblies. ** Chart showing the number of contigs in EST assemblies generated with GsAssembler using minimum overlap identity levels between 75 and 99% (see Methods). Assemblies were carried out from four data sets; *N. sylvesteris *(green), *N. tomentosiformis *(blue), *N. tabacum *(red) and a hybrid set of *N. sylvesteris * and *N. tomentosiformis * (brown) sequences.

An assembly strategy was adopted to maximize contig length while attempting to separate homeologous genes within *N. tabacum*. To identify optimal parameters, a set of assemblies were conducted using four EST datasets generated with 454 sequencing chemistry: *i- N. tabacum* ESTs, *ii- N. tomentosiformis* ESTs, *iii- N. sylvesteris * ESTs and *iv-* a combined dataset of the *N. sylvesteris * and *N. tomentosiformis* ESTs (to represent a synthetic polyploid transcriptome). The contigs generated from the four data sets were analyzed for a minimum overlap identity parameter set to a range of values between 75% and 99% (Figure 1). The *N. tabacum* ESTs and the synthetic polyploid data set produced a similar profile. An increase in the number of contigs was observed using a 97% identity setting (Figure 1). Unlike the *N. tabacum* and combined assemblies, the number of contigs in the individual *N. sylvesteris* and *N. tomentosiformis* assemblies did not increase at this level (Figure 1). The result suggested that 97% was the optimal identity threshold that could be used to separate homeologous sequences in the *N. tabacum* data set and homologous sequences in the combined data set without having a detrimental impact on the *N. sylvesteris* and *N. tomentosiformis* assemblies.

All four data sets showed an increase in the number of contigs when an identity setting of 99% was used. This level was considered too stringent as it was likely to be separating sequences based on sequencing errors (Figure 1). The assemblies based on an identity of 97% therefore provided the best data sets for subsequent analysis. This was further supported by manual inspection of contigs from the *N. tabacum* assemblies using the Tablet assembly viewer [32]. Manual inspection confirmed that contigs with more than 3 SNPs per 100 bp generated in the 95% identity assembly had correctly been separated into two contigs in the 97% identity assembly (data not shown).

Relative to the number of contigs in either individual assembly, the total number of contigs in the combined *N. sylvesteris* and *N. tomentosiformis* assembly was reduced, suggesting the collapse of orthologous sequences in the combined assembly. The lower number of contigs for the *N. tabacum* assembly compared with the *N. sylvesteris* and *N. tomentosiformis* assemblies may be partially explained by the higher number of sequences included in these assemblies. Increasing the number of *N. tabacum* reads with additional sequencing libraries, not included in this study, did indeed increase the number of contigs in the assembly (data not shown). However, a more likely explanation for the lower number of contigs in the *N. tabacum* and combined assemblies was there being no, or very low sequence polymorphisms between the orthologous genes of the ancestral parents, making it impossible to separate them during assembly at 97%.

To investigate the percentage of homeologs that were collapsed during the assembly process, reads from *N. tabacum*, *N. sylvesteris * and *N. tomentosiformis* were mapped onto the *N. tabacum* assembly produced with the 97% identity setting. Sequence polymorphisms could not be detected between the reads from the three species for 67% of the *N. tabacum* contigs (9718 contigs), indicating that sequences for a large portion of the assembly, were likely to have collapsed. This also meant that these sequences were not amenable to subsequent phylogenetic analysis. The remaining 33% of the sequences showed SNPs between the *N. tomentosiformis* and *N. sylvesteris* orthologs. When mapped against the *N. tabacum* assembly, a low number of *N. tabacum* sequences (3.4%) showed SNPs either supporting the possibility of collapsing of homeologous sequences in the assembly, or sequencing errors.

The three separate assemblies for *N. tabacum*, *N. sylvesteris* and *N. tomentosiformis* transcripts were further assembled using GsAssembler. In order to cluster the homeologous and homologous sequences across all three assemblies, the identity parameter of this combined *Nicotiana* assembly was set to the lower stringency level of 95%. PhygOmicss, a custom data processing pipeline was developed in order to carry out a phylogenetic analysis of the sequences for the entire transcriptome. The 17,220 clusters generated from the combined *Nicotiana* assembly were processed through the pipeline which selected 7974 clusters containing at least one contig of each individual species for further analysis. Alignments were then extracted and filtered by the length of the sequence overlap (minimum of 100 bp) and the average alignment percentage identity (minimum of 75%).

The consensus sequences for each of the clusters were annotated based on homology using BlastX [33]. Searches were conducted against four datasets and annotation results are summarized in Table 1. As expected, the *Nicotiana* clusters demonstrated the highest number of matches against the tomato gene model dataset (ITAG2). InterProScan [34] was used to perform a protein domain analysis on 13,504 of the clusters, 6913 of which had been annotated using the BlastX method previously described.

**Table 1:** **Annotation results from combined *Nicotiana *assembly based on homology **

**Database**	**Number annotated (%)**
GenBank NR [35]	14,102 (81.9%)
Swissprot [36]	9131 (53.0%)
TAIR9 [37]	13,219 (76.8%)
ITAG2 tomato	14,711 (85.4%)
InterPro [34]	13,504 (78.4%)

### Topology analysis of *Nicotiana* genes

The combined *Nicotiana* assembly was used to construct a set of phylogenetic trees for each cluster of sequences. Phylogenetic trees were constructed for 14,344 *Nicotiana* clusters that also contained at least one possible *Solanum lycopersicum* homolog as an out group. Bootstrapping analysis and filtering of these clusters (see Materials and Methods) identified 968 as containing either a single *N. tabacum* sequence, or two *N. tabacum* sequences along with the *N. tomentosiformis*, *N. sylvesteris* and *S. lycopersicum* sequence members.

**Figure 2:**
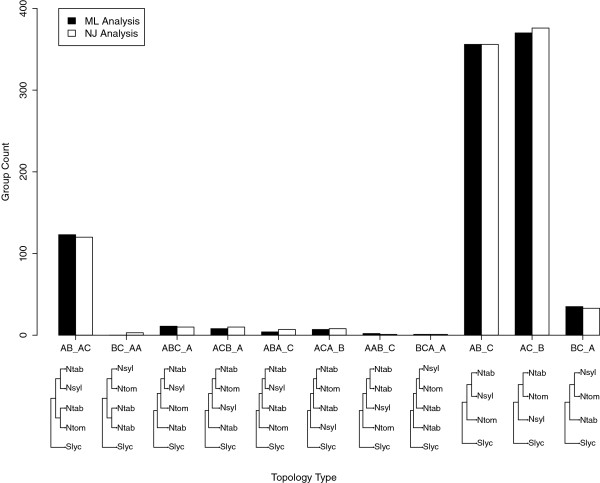
**Phylogenomic analysis of *Nicotiana *gene clusters. ** Bar chart showing the number of *Nicotiana* genes that were present in a set of pre-defined phylogenetic tree topologies. Genes from the *N. tabacum*, *N. sylvesteris * and *N. tomentosiformis * assemblies were clustered and phylogenetic trees for each cluster were generated by Maximum Likelihood (ML; black bars) and Neighbour Joining (NJ; open bars) methods using *S. lycopersicum* as an out-group. The different tree topologies are shown along the x-axis with *N. tabacum* (A; Ntab) *N. sylvesteris * (B; Nsyl), *N. tomentosiformis * (C; Ntom) and *S. lycopersicum * (Slyc) genes represented in text and/or dendrogram form.

Neighbor Join (NJ) and Maximum Likelihood (ML) methods were used to build phylogenetic trees for each of the 968 clusters (Additional files 1 and 2). The topologies of these trees were grouped into 11 categories. The distribution of the results in these 11 categories was similar between the NJ and ML methods (Figure 2). Approximately 10% of the clusters contained two *N. tabacum* sequences, each of which could be associated with the respective *N. sylvesteris* and *N. tomentosiformis* sequences. This topology would be expected if both the T and S homeologs had been maintained and expressed by the plant following polyploidization (AB_AC; Figure 2). The majority (approximately 90%) of clusters, however, contained only a single *N. tabacum* sequence the majority of which could be associated with either the *N. sylvesteris* (AB_C) or *N. tomentosiformis* (AC_B) sequence (Figure 2). Given that these clusters contained genes where SNPs existed between the parental homeologs, reducing the likelihood of collapse of the sequences during assembly, the abundance of this latter topology is most likely explained by either gene subfunctionalization (when the absent gene is not expressed in the tissue analyzed), or gene loss/nonfunctionalization.

**Figure 3:**
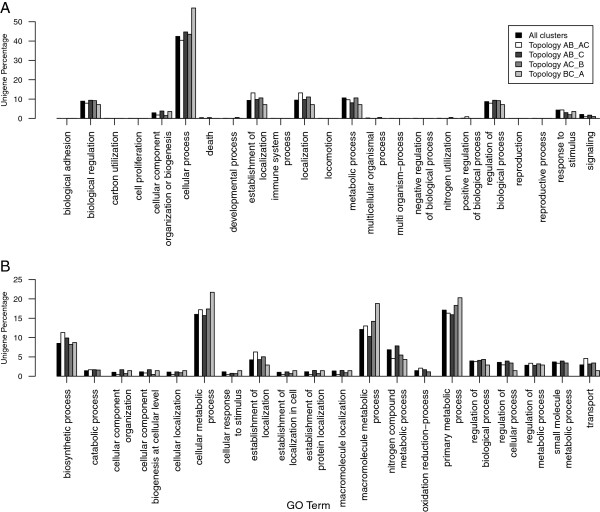
**Gene Ontology analysis of *Nicotiana *gene clusters. ** Plot showing the percentage of *Nicotiana * gene clusters annotated with level 2 (**A**) and level 3 (**B**) Biological Process Gene Ontology terms for all gene clusters and each of the main phylogenetic tree topologies (AB_AC, AB_C, AC_B and BC_A). Bars are coloured according to topology group (see inset key for identification).

Gene Ontology (GO) analysis of the most abundant topologies from the *Nicotiana* data (AB_AC, AB_C, AC_B and BC_A) was performed [38]. Figure 3 shows the representation of GO Biological process terms (levels 2 and 3) for these topologies. No significant differences between AB_AC, AB_C and AC_B were observed relative to the biological process categories. The same was true when comparing AB_C and AC_B topologies for cellular component and molecular function categories compared to the global list of the combined *Nicotiana* consensus sequences. Three percent of the trees showed an unexpected topology with the *N. sylvesteris* and *N. tomentosiformis* sequences being more closely related to each other than to the *N. tabacum* sequences (BC_A; Figure 2). While false clustering of an *N. tabacum* paralog with the *N. sylvesteris* and *N. tomentosiformis* sequences is the most likely explanation for the anomaly, GO analysis showed significant overrepresentation (*P* < 0.05) in some categories, such as pathogenesis signaling (see Additional file 1), for these clusters, suggesting that they may contain genes with an interesting evolutionary history. However, it should be noted that this set of clusters contained fewer than 50 members.

### Identification and expression analysis of *Nicotiana tabacum* homeologs

Estimations of gene expression levels were calculated based on the number of sequence reads and used to compare gene expression levels for the three different *Nicotiana* species. For the 9718 *N. tabacum* transcript clusters (67% of total number of clusters) where there were no reliable inter-specific SNPs that could be used to identify the ancestral origin, only 2171 transcript clusters contained five or more reads for each of the three *Nicotiana* species. The same expression levels (R < 7, see Materials and Methods) across all three species were observed for 83.6% of these genes. Among the remaining differentially expressed genes, the most frequent category was *N. tabacum* genes (with no distinction between homeologs) overexpressed in comparison with *N. sylvesteris* and *N. tomentosiformis* (4.7%), followed by *N. tabacum* genes with similar expression to the *N. sylvesteris* homolog (4.5%) and *N. tomentosiformis* homolog (4.2%). Only 0.7% of the *N. tabacum* genes were expressed at a lower level when compared with both parental sequences. The rest of the transcripts (2.3%) showed variable trends relative to differential expression between all the transcripts (for example, over-expressed compared to one of the parents and the contrary when compared to the other parent).

741 of the 968 gene clusters described above contained 975 *N. tabacum* consensus sequences (5.3% of the total *N. tabacum* sequences) that could be assigned ancestral origin based on the phylogenetic trees. 482 of these sequences were assigned to *N. sylvesteris* (S) origin and 493 sequences were assigned to *N. tomentosiformis* (T) origin. A total of 103 gene clusters (10.6% of the topology analyzed clusters, 0.6% of the total) showed differential expression (R > = 7): 51 gene clusters (0.3% of the total) for *N. tabacum* S-homeologs (ie, AB_C), of which 22 clusters (0.1% of the all clusters) were overexpressed in comparison with *N. sylvesteris* homeologs. Similar results were observed for *N. tabacum* T-homeologs (from topology AC_B); 52 clusters (0.3%) showed differential expression (R > 7) and of them 8 *N. tabacum* clusters (0.05%) were overexpressed in comparison with *N. tomentosiformis*.

**Figure 4:**
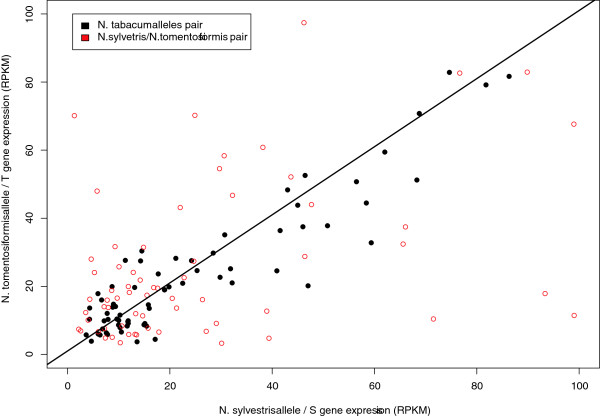
**Expression level of *Nicotiana *gene pairs. ** Scatter plot showing the expression level (RPKM) for the *N. sylvesteris * / S genome gene (x-axis) versus *N. tomentosiformis * / T genome gene (y-axis) for homologous gene pairs (open red circles) and homeologous *N. tabacum * gene pairs (closed black circles). Solid black line across diagonal represents no difference in gene expression level between species.

Of the 274 AB_AC gene clusters containing two *N. tabacum* sequences, 77 clusters were selected where the consensus sequences were built with at least 5 reads. Figure 4 shows scatter plots comparing expression levels of the homeologous and homologous gene pairs for these gene clusters.

Differential expression (R > = 7, see Materials and Methods) was observed for 27.3% of the *N. tabacum* homeologous gene pairs (21 of the 77 gene clusters, 2.2% of the topology analyzed clusters, 0.1% of the total transcribed genes). In comparison to the parental homeologs, 3.9% of the T-genes (3 clusters) and 11.7% of the S-gene (9 clusters) were over-expressed. Only 3.9% of the genes demonstrated differential expression when comparing T-genes with S-genes in *N. tabacum* (3 clusters, in one of which S-gene expression was higher than the T-gene). In comparison, 22.7% of the homologous genes in this set were differentially expressed between the *N. tomentosiformis* and *N. sylvesteris* samples. A more consistent level of gene expression between *N. tabacum* homeologs was also indicated by the Pearson correlation coefficient, which was higher between these genes than between the *N. sylvesteris* and *N. tomentosiformis* homeologs (*R*^2^ values of 0.93 and 0.82, respectively). The increased level of differential expression between the homologous genes pairs may simply reflect that the comparison was conducted using independent samples and may be due to experimental/biological variation. The homeologs comparison was conducted between genes from the same *N. tabacum* sample. However, the data clearly suggests that in the vast majority of cases when both *N. tabacum* homeologs are expressed in the same tissue (such as the leaf tissue analyzed here) there is little difference in expression on a transcriptional level.

Given the small number of homeologous gene pairs showing differential expression, the function of these genes were analyzed. Genes with higher expression of the S homeolog showed over-representation of GO terms associated with the biological processes for proteolysis, protein folding and aldehyde metabolism. Genes with higher expression of the T homeolog showed over-representation of GO terms associated with the biological processes for oligopeptide transport and translation.

### Non-synonymous and synonymous site substitution rates between *N. tabacum*, *N. sylvesteris* and *N. tomentosiformis* species

Comparison of expression in only a single tissue/organ is too limited to differentiate between redundancy and subfunctionalization. A more extensive expression study might provide the ability to distinguish between these two evolutionary processes. Cases of neofunctionalization, however, could be distinguished by a comparison of the gene sequences in the current data set.

Changes to a gene sequence resulting in an altered protein sequence potentially alters the function of that gene. A comparison of the rate of synonymous (Ks) and non-synonymous (Kn) nucleotide substitutions provides insights into the evolutionary history of a gene [39,40]. Genes showing a low rate of non-synonymous substitutions are likely to have undergone strong selective pressure to be conserved more faithfully and thus their function maintained. Genes showing a relatively high level of non-synonymous substitutions are likely to have undergone positive selection and possible neofunctionalization [41].

**Figure 5:**
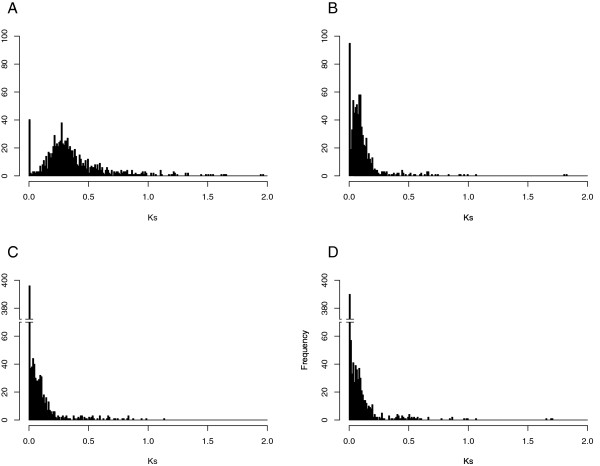
**Nucleotide substitution rates in *Nicotiana *genes.** Frequency histograms showing the rate of synonymous nucleotide substitutions (Ks) in orthologous genes between *N. tabacum * and *S. lycopersicum * (**A**), *N. sylvesteris * and *N. tomentosiformis* (**B**), *N. tabacum* and *N. sylvesteris * (**C**) and *N. tabacum * and *N. tomentosiformis * (**D**).

To estimate the rate of synonymous (Ks) and non-synonymous (Kn) nucleotide substitutions, an analysis was carried out using clusters of genes selected when topology analysis suggested that either one or both *N. tabacum* homeologs were maintained and could be assigned to T or S origin, (mainly topologies AB_AC, AB_C, AC_B and BC_A, Figure 2; 787 clusters, 3251 sequences). The ratio between Kn and Ks (ω) for each pair of sequences was also calculated. Figure 5 shows the distribution of Ks for sequence pair comparisons between the *Nicotiana* species. For reference, a comparison between *N. tabacum* and *S. lycopersicum* genes is also shown (Figure 5A). This older divergence event showed a higher rate of Ks relative to the comparisons between the *Nicotiana* species. Peak distribution of Ks values were approximately 0.27, compared to approximately 0.09 for the *N. sylvesteris* and *N. tomentosiformis*. Ks values were even lower for the *N. tabacum* to *N. sylvesteris * and *N. tomentosiformis* comparisons (Figure 5). The high number of sequence pairs with a Ks value < 0.01 in the comparison between *N. tabacum* and *N. sylvesteris * (397), or *N. tomentosiformis* (390) as compared to the number between *N. tabacum* and *S. lycopersicum* (39) or between *N. sylvesteris * and *N. tomentosiformis* (96) suggests that a high percentage of the *N. tabacum* genes have not diverged from their ancestral sequences (Figure 5).

**Figure 6:**
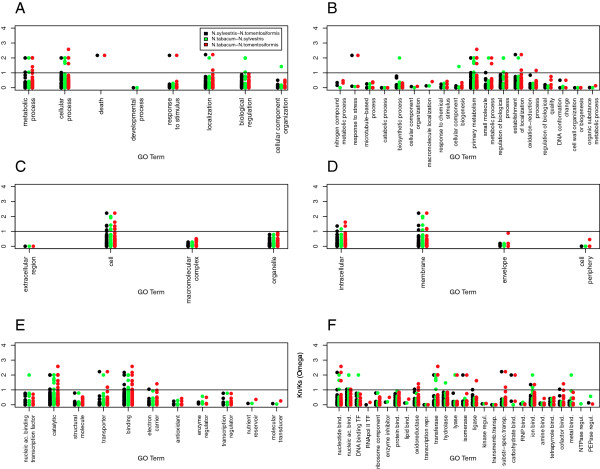
**Evolutionary rates in *Nicotiana *genes from different Gene Ontology groups. ** Non-synonymous: synonymous nucleotide subtraction ratio (ω) values for *Nicotiana * genes separated according to their Level 2 (**A**) and Level 3 (**B**) Biological Process, Level 2 (**C**) and Level 4 (**D**) Cellular Component and Level 2 (**E**) and Level 3 (**F**) Molecular Function Gene Ontology annotations. Omega values for comparisons between homologous *N. sylvesteris * and *N. tomentosiformis * gene pairs (black circles), *N. tabacum * and *N. sylvesteris * gene pairs (green circles) and *N. tabacum * and *N. tomentosiformis * gene pairs (red circles) are shown.

Analysis of the Kn/Ks ratio demonstrated that the majority of genes had an ω value lower than 1, suggesting that few genes had undergone positive selection during the evolution of *N. tabacum* (assuming the limitations of Kn/Ks for the gene positive selection studies [42]). Only 3% of the clusters showed positive selection associated to *N. sylvesteris* and *N. tomentosiformis* homeologs and the correspondent homeologs (*N. tabacum* S- and T-genome), but no positive selection between the *N. tabacum* and the parental homeologs pairs (*N. tabacum* S-genome*/N. sylvesteris* or *N. tabacum* T-genome*/N. tomentosiformis*). The GO annotations associated with the small number of genes that were undergoing positive selection showed a similar distribution across the three species and corresponded to the more representative GO term categories such as metabolic process, cellular process, cell, catalytic activity and binding (Figure 6). Genes with an ω > 1 for the *N. sylvesteris* and *N. tabacum* (T-genome) homolog pairs (22 clusters) showed overrepresentation of the level 2 biological process ontologies: biological regulation, cellular component organization and regulation of a biological process. Examples of these genes include cluster 00509 (similar to *Arabidopsis thaliana* APL transcription factor involved in biological regulation) and 02926 (NAC domain protein involved in biological regulation). The 23 genes with an ω *>* 1 for the *N. tomentosiformis* and *N. tabacum* (S-genome) homolog pairs showed over-representation of the GO terms cellular process, developmental process and metabolic process. This included clusters 04302 (similar to *Arabidopsis thaliana* E3 ubiquitin-protein ligase SINAT2 involved in some developmental process), 04210 (Tyrosyl-tRNA synthetase) and 02320 (similar to cell division protein ftsy).

Within the set of genes analyzed, there were no instances of homeologous genes from *N. tabacum* demonstrating positive selection (ω > 1). A comparison between the respective *N. tomentosiformis* and *N. sylvesteris* homeologs also did not show any instances of positive selection. This absence of positive selection suggests that the majority of positive selection represented in the gene set analyzed occurred following the divergence of the two ancestral species rather than since the formation of *N. tabacum*. It also suggests that the rate of neofunctionalization in *N. tabacum* has been relatively low.

## Discussion

### Polyploid species sequence assembly

Using a next generation sequencing approach, leaf transcriptome sequence data was generated for the allotetraploid *N. tabacum* and its progenitor species *N. sylvesteris* and *N. tomentosiformis*. These sequences were assembled into species specific sets of unigenes and then further combined into a consensus set of clusters for the three species. The process of assembly revealed that default parameters of sequence assemblers were probably not stringent enough when working with sequences originating from polyploid species. Sequencing errors, such as homopolymer length issues associated with pyrosequencing, can further confound this problem by potentially masking low polymorphism content between homeologs. Other sequencing technologies, such as Illumina, may not be impacted by this homopolymer problem, but read length may be a limiting factor given the requirement that a single read must contain at least one polymorphism per overlapping region. These factors should be taken into consideration for any future assembly attempts on polyploid species and the methodology applied for the assembly of an allopolyploid transcriptome in this study could be useful for guiding future genome assembly work in polyploidy.

Additionally, the number of collapsed homeologs was estimated in *N. tabacum* assembled transcripts (using the 97% identity assembly) based on SNPs shared with *N. sylvesteris* or *N. tomentosiformis* reads. In this analysis, only 3.4% of *N. tabacum* transcripts were polymorphic and shared SNPs with the parental transcripts. This methodology cannot be applied for transcripts lacking SNPs in the transcript fragment analyzed (67% of the transcripts). More information could be obtained by deeper transcriptomic sequencing (more mapped sequences and more reliable SNP calling), use of longer sequences (increasing the possibility to find a parent relative SNP) or genomic DNA sequencing (where the intron sequencing, being more diverge region, could increase the number of parent relative SNPs).

### Homeologous gene fate in *Nicotiana tabacum*

Based on the leaf transcriptome data for the *Nicotiana* species generated in this study, a pipeline was developed to carry out a phylogenetic analysis on a genomic scale. The PhygOmicss pipeline works on a single transcriptome set, but can be applied to transcriptomic data from multiple tissues/organs, or gene models from genomic sequence data.

The majority of the *N. tabacum* transcripts (69%) did not show any polymorphisms with the parental sequences, making it impossible to distinguish the homeologous genes and excluding the possibility of neofunctionalization in these genes. Additionally the expression analysis of clusters with genes expressed above background level (more than 5 reads), revealed that the expression of a majority of these genes was not changed (83.6% of genes in clusters; 57.7% of the total transcribed genes) between these three species. With this level of conserved expression, the possibility of subfunctionalization is low.

A more specific topology analysis with the newly developed PhygOmicss pipeline revealed that in *N. tabacum* transcripts where homeologous genes can be differentiated there was evidence for the presence of only a single homeolog (90% of gene clusters, 6% of the total transcribed genes). Given that the data is transcriptomic, it is not possible to distinguish between gene loss and subfunctionalizaton. Tissue-specific gene silencing [14] provides one possible mechanism of gene subfunctionalization and may partially explain the pattern observed in the *Nicotiana* topologies. An analysis of a broader set of tissues might resolve the question and increase the chance of detecting expression differences in any individual genes. However, studies in other polyploid plants suggest that only a small number of genes display tissue specific gene silencing. For example, a similarly low level of gene silencing (around 1-5%) was estimated in both synthetic allotetraploid wheat [8] and synthetic cotton [10], and results from gene expression analysis of *Tragopogon miscellus* showed a similar trend (3.4%) [43]. Even lower estimates of silencing were suggested from experiments with an early allotetraploid formed by the hybridization of *Arabidopsis thaliana* and *Cardaminopsis arenosa* (> 0.4%) [7].

Based on the distribution of topologies and the relative expression level of homeologous genes, there was little evidence to suggest preferential loss, or transcriptional silencing of genes from one or other progenitor genomes from the sub-set of *Nicotiana* sequences that this analysis could be completed on. This is in contrast to the apparent preferential loss of repetitive sequences from the T genome in *N. tabacum*, as shown in a recent study also using 454 sequencing in these *Nicotiana* species [27]. Previous studies in other allotetraploids have shown preferential expression of homeologous genes. For example, there is evidence of preferential expression of the D genome in cotton [44]. Differential expression was shown for 22% of homeologous genes pairs in the 40 generation-old allotetraploid *T. miscellus*[13], similar to the 27% of *N. tabacum* genes observed in this study. It should also be noted that genes expressed in the leaf tissue at a very low level may have been missed in the transcriptome sets, particularly since clusters with less than 5 sequence members were removed from the analysis. As such, increasing the sequence depth might reveal more differentially expressed homeologous genes, but it is unlikely that this will increase the contribution of subfunctionalization extensively.

With the caveat that this study was based on a subset of genes identified in the leaf transcriptomes of *Nicotiana* species, the data would suggest the expression of homeologous genes is mostly conserved between *N. tabacum* and its parent relatives and supporting the hypothesis of gene dosage compensation [15,45] reported previously in other species [46]. This level may be over-estimated as the transcriptome was sampled in only one tissue type, thus reducing the possibility of observing subfunctionalization. However, based on the levels observed in other species [7,8,10,43] subfunctionalization is unlikely to account for a large proportion of genes.

There is also limited evidence of neofunctionalization having occurred in *N. tabacum*, based on comparison of the homeologous and homologous gene sequences. Indeed, no genes could be identified as undergoing positive selection in *N. tabacum* that did not also show the same response between *N. sylvesteris* and *N. tomentosiformis*. This suggests that these differences may have predated the formation of tobacco. Again, the apparent low level of neofunctionalization may be explained by only having sampled the leaf transcriptome. Sequencing transcripts from other tissues, perhaps more specifically involved in secondary metabolite synthesis, may increase the likelihood of identifying genes showing positive selection in tobacco; two such examples are trichomes [47] or roots, where alkaloids, including nicotine, are synthesized [48].

In addition to an increased spatial and temporal coverage of the transcriptome for the *Nicotiana* species covered in this study, it would be interesting to compare the proportion of subfunctionalization and neofunctionalization in tobacco with an older *Nicotiana* allotetraploid species, such as *Nicotiana nesophila* (dated approx. 4.5 Myr old), or *Nicotiana benthamiana* (dated > 10 Myr old) [20]. Similarly, a comparative analysis of allele selection between wild and cultivated *N. tabacum* varieties might provide insight into the role of homologous genes in the species’ domestication process. Gene duplication plays an important role in the successful transition of a wild species into its cultivated relatives, as shown for several wheat loci [49]. Indeed there are also examples for duplicated genes from diploid species playing an important role in domestication, including *GRAIN INCOMPLETE FILLING 1* (*GIF1*) and the cell wall invertase *OsCIN1* in [50].

## Conclusions

This study represents the first time that a phylogenetic analysis of the tobacco genes has been carried out on a genome scale to further elucidate the complex evolutionary history of the species. Transcriptome assembly for polyploid species possesses the intrinsic difficulty of homeolog collapse. 67% of the *N. tabacum* assembled transcripts lack any polymorphism that can be used to elucidate the sequence origin. Read depth, read length and use of more variable regions such as introns will be critical to dissecting these genes.

There was evidence of a general maintenance of the expression levels between *N. tabacum*, *N. sylvesteris* and *N. tomentosiformis* homeologs. Despite the conservation of transcriptomic levels in tobacco, there was little evidence for the occurrence of neofunctionalization, suggesting that, at 0.2 Myr old, tobacco may be too young evolutionarily and that this is a more common fate for duplicated genes in older polyploidy species. There may, however, be particular interest in comparing cultivated with more primitive varieties using the method developed here in order to identify the genes selected during the domestication of tobacco. The low level of neofunctionalization may make such an analysis easier.

## Methods

### Plant material

*Nicotiana tabacum* (cv. K326), *N. sylvesteris* and *N. tomentosiformis* plants (in-house accessions available at ATC) were grown in a glass house on soil (Levingtons M2) under 16 h: 8 h light dark cycles. Fully expanded green leaves were harvested from 3–4 month old plants, snap frozen in liquid nitrogen and stored at −80°C.

### Transcriptome sequencing

Leaf samples were ground under liquid nitrogen and total RNA was extracted using Trizol (Invitrogen, Paisley) and purified with RNeasy spin columns (Qiagen, Crawley, UK) according to the manufacturer’s instructions. mRNA was isolated with a Dynabead kit and quantified using a RiboGreen assay (Invitrogen, Paisley, UK).

Sequencing libraries were generated from 200 ng mRNA using the cDNA Rapid Library preparation method and sequenced on the GS FLX Ti according to manufacturer’s instructions (Roche, Burgess Hill, UK).

### Sequence assembly and annotation

Test assemblies of the *N. sylvesteris* ESTs were carried out with default parameters using the assemblers GsAssembler (version 2.5.3; Roche), MIRA (version 3.0.5) and CAP3 (version 10/15/07).

Sequence assembly was performed on an SGN server (Red Hat Enterprise Linux Server release 5.4, CPUs: 48 cores, RAM: 256 Gb). GsAssembler version 2.5.3 was used with the option cDNA enabled. Seven different assemblies were created with identity values of 75, 80, 85, 90, 95, 97 and 99 in a minimum overlap length of 40 bp for each of the samples plus an extra sample with half of a 454 run of *N. sylvesteris* and half of a run of *N. tomentosiformis*. Contigs were selected using a perl script with a length cutoff value of 40 bp. The reassembly of the contigs for each sample was performed with the same software using an identity percentage value of 95. Contigs with a length of 2000 bp or longer were reassembled with CAP3 [51].

The collapse of homeologous sequences was evaluated by remapping all the Nicotiana reads with Bowtie [52] using the *N. tabacum* transcriptome assembly with 97% minimum identity as reference sequence. The mapping file in sam format was filtered and SNPs were called using Samtools and Bcftools [53]. SNP files were loaded into a generic Postgres database to perform a simple full outer join search.

Sequences were annotated using the basic local alignment search tool (blastx [35]) with the databases: GenBank nr [33], Swissprot [35] and TAIR9 [36] and 1e-20 e-value cutoff. Proteins were predicted using EstScan [54] and domain annotation was performed using InterProScan [34]. Gene Annotations were analyzed with the Bioconductor module goProfile [38].

### Tree topology analysis

Tree topology analyses were performed with the PhygOmicss pipeline (manuscript in preparation). Sequence alignments were extracted from the assembly ace file with a Perl script integrated into the PhygOmicss pipeline. *Solanum lycopersicum* sequence homeologs were assigned based on the BLAST [37] results of the consensus sequence of the *Nicotiana* alignments with the Tomato Gene Model ITAG2 dataset. Only matches with alignment lengths of more than 100 bp and a nucleotide identity percentage of at least 70% were selected as homeologs. They were aligned with the Nicotiana sequences using ClustalW [55], Mafft [56] and Muscle [57] programs as an integrated part of the PhygOmicss pipeline. Mis-alignments were quantified using the global alignment length and the identity percentage average of each alignment, discarding the alignments with lengths shorter than 100 bp and identity percentages lower than 75%. ClustalW was run with non-default parameters (see Additional file 2 for the configuration parameters for the pipeline) as the preferred alignment program based on a minimum number of mis-alignments (data not shown). A pruning Perl script that is part of the pipeline was used to select closely related sequences for each alignment, based on a maximum alignment score (manuscript in preparation). Alignments that did not include members of one of the selected species (*N.tabacum, N.sylvesteris, N.tomentosiformis* and *S. lycopersicum*) were discarded. Phylogenetic trees using *S.lycopersicum* sequence as out-group were constructed with Phylip [58] following two methods: Neighbor-joining [59] and Maximum Likelihood [60]. A bootstrapping with 1000 replicates was also performed for each tree. Trees containing nodes with low bootstrap support (under 60%) were discarded. 968 isotigs (5.6% of the total isotigs produced in the assembly) produced trees that were able to be analysed based on these parameters.

Topology comparisons were performed using a Perl module Bio::Tree::Topology as an element of the pipeline (see additional file 3 for the PhygOmicss configuration file). This module compares if the tree obtained is the same after the replacement of the tree leaves (contig ids) with the sample source (species) and the branch length with an equivalence value (0.01 as length cutoff value).

### Homeolog identification

Homeolog identification was performed by leaf species identity in a neighbor comparison using the PhygOmicss pipeline. Homeolog assignment was performed based in the closest parental homolog in the tree structure. A cutoff value of 90% identity and 60 bp length in the alignment between the candidate homeologs and the reference sequence were used.

### Expression analysis

Expression analysis was performed parsing the assembly .ace files using a Perl script. Read count and RPKM calculation [61] was performed with Perl scripts available at the Solgenomics GitHub page (https://github.com/solgenomics/sgn-home/tree/master/aure/scripts/phylo/PhygOmicss). The R statistical differential expression value was calculated as described by Stekel [62].

### Non synonymous-synonymous analysis of *N. tabacum* homeologs

Non-synonymous to synonymous analysis was performed using Codeml from the PAML software package [63] through the PhygOmic pipeline. CDS sequences used with Codeml were predicted with GsAssembler (longest 6 frame method). The results were parsed using a Perl script. Clusters with pairs with omega values = 99 were removed from the analysis. A close inspection of majority of them revealed sequences with an identity of 100% where the Kn/Ks ratio was 0/0.

## Abbreviations

Myr: Million years; RPKM: Reads per kilobase per million of reads; NGS: Next generation sequencing.

## Competing interests

The authors have no competing interests.

## Authors' contributions

AB, KDE and LAM conceived of the study. All authors were involved in the writing and editing of paper. Sequencing was carried out in the laboratory of KDE. PhygOmics pipeline development and bioinformatics analysis was carried out by AB. All authors read and approved the final manuscript.

## Supplementary Material

Additional file 1 Functional annotation table for clusters BC_A and AB_AC. Click here for file

Additional file 2 Configuration file for PhygOmicss pipeline analysis of the Nicotiana transcriptome. Click here for file

Additional file 3 Clusters sequence composition, topology and functional annotation using Blast hits. Click here for file
